# Gender roles, sociosexuality, and sexual behavior among US Black women

**DOI:** 10.1080/21642850.2014.882236

**Published:** 2014-02-17

**Authors:** Naomi M. Hall, Latrice C. Pichon

**Affiliations:** ^a^ Department of Psychological Sciences, Winston-Salem State University, Coltrane Hall 203, Winston-Salem, NC27110, USA; ^b^ Division of Social and Behavioral Sciences, School of Public Health, The University of Memphis, Memphis, TN, USA

**Keywords:** Black women, sociosexuality, gender roles, sexual behavior, HIV

## Abstract

This study examined the relationship between gender roles and sociosexuality (an individual difference variable describing attitudes about sexual permissiveness and promiscuity), and their predictive pattern of HIV-related sexual risk behaviors. A geographically diverse sample of 275 adult, heterosexual Black women (mean age = 33.60 years), participated in a self-administered survey. Significant relationships were found between feminine traits and sociosexuality, and between sociosexuality and four of the five risky sexual behavior variables. Neither masculine nor feminine gender roles were related to any risky sexual behavior variables. Sociosexuality emerged as an important correlate that requires further exploration of its relationship to the attitudes and behaviors of Black women, and its potential relationship to HIV risk-related sexual behavior. The need for more attention to psychosocial variables, and consideration of context, cultural norms, and values is discussed as an important undertaking in order to garner an accurate picture of sexual risk behavior.

## Introduction

The HIV pandemic has become increasingly feminized and centralized in communities of color with rates growing exponentially (Centers for Disease Control and Prevention [CDC], 2013; HIV Prevention Trials Network [HPTN], 2012). African American^1^ women have been disproportionately infected and affected by HIV/AIDS since the beginning of the epidemic and continue to represent the majority of new HIV infections and AIDS diagnoses among women in the USA (Kaiser Family Foundation, 2012). In 2010, the estimated rate of new HIV infections among Black women in the USA was 20 times higher than for White women, and almost 5 times higher than Latinas (CDC, 2013). According to the same source, the large majority of those infected (87%) are thought to have acquired HIV through heterosexual intercourse.

According to the Centers for Disease Control and Prevention, at some point in their lifetime, an estimated 1 in 32 African American women will be diagnosed with HIV (CDC, 2011). With such elevated incidence and prevalence rates, the need to focus on sexual behaviors, as well as sexual attitudes and their contribution to HIV risk among heterosexual African American women is imperative. Two important contributors to sexual attitudes and behavior are gender roles and sociosexuality. The purpose of this study is to examine the relationship between gender roles and sociosexuality, and to explore their predictive patterns of sexual risk behavior among adult, heterosexual African American women in the USA.

### Gender roles

Gender roles are beliefs regarding the specific tasks, personalities, and behaviors that men and women are “expected” to embrace (Littlefield, 2003; Nguyen et al., 2010). Gender roles influence and sometimes define the behavior and the interpersonal relationships of men and women (Ehrhardt & Wasserheit, 1991). Studies done exclusively with Black women have found that they tend to hold more egalitarian gender ideologies than White women or Latinas (Harris & Firestone, 1998). Additionally, researchers found that Black women are more likely than other groups of women to hold expressive (i.e. traditionally feminine) *and* instrumental (i.e. traditionally masculine) gender ideologies simultaneously (Bowleg, Belgrave, & Reisen, 2000). Instrumental traits are those that tend to emphasize traditional masculine characteristics, such as assertiveness and independence. Expressive traits are those that are characterized as traditionally feminine, such as being sensitive to others' needs and emotions. Women who score high on both the instrumental and expressive scales have been traditionally characterized as androgynous. Binion (1990) provided a perspective on “psychological androgyny” by relating it to the history of slavery and institutional discrimination that dictated that Black women work outside the home to support themselves and their families, unlike women of other groups. This unique socio-political history would suggest that African American women have used androgynous characteristics as both a coping and survival strategy. In fact, research has also shown that androgynous characteristics are associated with more positive psychological functioning of African American women (Cooper, Guthrie, Brown, & Metzger, 2011). Even though women may need to utilize more instrumental traits in the workplace, cultural and relational values may dictate that women must still maintain expressive traits within the household and relationship. This may suggest that one of the prevailing cultural scenarios for Black women is that they should be nontraditional in the workforce setting, but traditionally feminine in their intimate relationships.

### Gender roles and sexual behavior

In the context of HIV/AIDS, gender-related dynamics are relevant to whether women will take an active or passive role in sexual situations including, but not limited to, initiating discussions with a sexual partner about safer sex practices, deciding whether and when safer sex practices will occur, using strategies to negotiate or assert power to protect their own health, or refusing to engage in risky sexual practices (Bowleg et al., 2000). Consideration of the socially and culturally defined gender role context is essential in understanding sexual attitudes, decision-making, and behavior (Amaro, 1995). Amaro argued that within heterosexual women's ascribed gender roles, sex is something that women give to men, leaving little room for women's acceptance of their own sexuality.

Studies show that women who adhere to traditional gender norms are more likely to engage in behaviors that increase their risk of contracting HIV (Campbell, 1995; Pleck, Sonenstein, & Ku, 1993; Sikkema, Wagner, & Bogart, 2000; Wingood & DiClemente, 1997). Bowleg et al. surveyed 125 African American and Latina women (mean age = 28.30 years, 47% African American) and found that lower (or inconsistent) condom use was significantly related to gender roles. A study surveyed 154 college students (mean age = 20.8 years, 76% White) and found that traditional gender role attitudes resulted in riskier sexual behavior such as casual sex and inconsistent condom use (Shearer, Hosterman, Gillen, & Lefkowitz, 2005). While the levels of traditional gender role adherence may be different for African American women than women of other races/ethnicities, the relationship with HIV risk behavior has been similar (Bird & Harvey, 2001; Bowleg et al., 2000; Jarama, Belgrave, Bradford, Young, & Honnold, 2007; Nguyen et al., 2010). In a qualitative study with 50 African American adolescents, stronger adherence to traditionally feminine ideology was related to the young women placing a higher value on emotional and relationship intimacy than the young men, which has been associated with engaging in riskier sexual behavior (Kerrigan et al., 2007). In another study with young African American women, results indicate that both egalitarian and traditional gender role attitudes were significantly related to having multiple partners and concurrent sexual partnerships (Leech, 2010). Much of the research conducted on gender roles and risky sexual behavior have not included an investigation into the potential impact of differential personality traits. Exploring the relationship between HIV behavioral risks and personality traits such as sociosexuality has potential implications for future research and practice-based interventions. Women may be more or less prone to engage in risky sexual behavior based on variance in certain underlying personality traits (Cooper, Agocha, & Sheldon, 2000). Without more focus on this connection, our understanding of key individual differences in HIV-related attitudes, beliefs, and behaviors is limited. We are interested in examining gender roles, engagement in risky sexual behavior, and an individual difference variable shown to be related to attitudes and behaviors around casual or uncommitted sexual relationships.

### Gender roles and sociosexuality

Gender roles have been shown to be significantly related to an individual difference variable known as sociosexuality (Fink, Brewer, Fehl, & Neave, 2007). Sociosexual is a term first used by Kinsey, Pomeroy and Martin (1948) to describe individual differences in attitudes about sexual permissiveness and promiscuity. The Sociosexual Orientation Inventory (SOI) identified a personality dimension, *sociosexuality*, believed to be associated with the extent to which one is willing to engage in sexual activity outside of a committed relationship (Simpson & Gangestad, 1991). Individuals considered *restrictive* typically insist on commitment and intimacy in a relationship prior to engaging in sex with a partner, having fewer lifetime partners, and take more time before having sex with someone (Simpson & Gangestad, 1991). Those who are considered *unrestrictive* may feel relatively comfortable engaging in sex without commitment or emotional closeness, report engaging in sexual activities at a younger age (Yost & Zurbriggen, 2006), are more likely to have concurrent sexual partners (Ostovich & Sabini, 2004; Yost & Zurbriggen, 2006) tend to express less investment, and have weaker affectional ties (Westerlund et al., 2010). Masculinity (or greater levels of instrumentality) has been found to be associated with an unrestricted sociosexual orientation; however, femininity (or greater levels of expressiveness) is associated with a restricted sociosexual orientation (Kinsey, Pomeroy, & Martin, 1948; Mikach & Bailey, 1999).

## Current study

A geographically diverse sample of heterosexual African American women participated in a quantitative study examining the sociocultural correlates of sexual behavior among adult heterosexual African American men and women. The authors were interested in exploring two research questions: (1) Is there a significant relationship between gender roles and sociosexuality? and (2) What is the relationship between gender roles and sociosexuality and five specific HIV-related sexual risk behaviors? Although gender roles have been explored in previous research, this study expands the current literature by examining its relationship with the personality variable, sociosexuality, among a geographically diverse, well-educated sample of heterosexual African American women. This is important because previous research primarily has been limited to sociodemographic factors associated with HIV risk among women who traditionally subscribe to expressive gender ideologies. Here we explore the variability in or continuum of Black women self-reporting their willingness to engage in casual, uncommitted sex.

## Method

### Participants

Participants were 275 self-identified heterosexual adult Black women from all four geographic regions in the USA. Heterosexual was operationally defined as no reported voluntary experiences with or sexual attraction towards the same gender. Participants ranged in age from 21 to 61 (*M* = 33.60 years, SD = 8.89). A small percentage indicated that they were born outside of the USA (8.1%), and the average number of years in the USA was 22.3. See Table 1 for sociodemographic information. Additional criteria for the study were that women had to be aged 21 and older, not knowingly pregnant, not knowingly HIV-positive, and had engaged in sexual activity (vaginal or anal) at least once in the past 12 months.

**Table 1.** Characteristics of heterosexual African American women (N = 275).

Variable	Entire Sample^a^ (*N* = 275)
*Mean Age (SD)*	33.60 (8.89)
*Geographic Region*	
Eastern	33%
Southern	22%
Midwestern	12%
Western	33%
*Educational level*	
High School Graduate	19%
College Graduate	33%
Advanced Graduate	48%
*Employed (%Yes)*	92%
*Income*	
Less than $25,000	14%
$25,001-$45,000	29%
$45,001-$65,000	31%
$65,001->$75,000	26%
*Current relationship status*	
Committed relationship	66%
Casual relationship	17%
Currently not involved in a relationship	17%
*Marital status*	
Married	32%
Single	57%
Other	11%
Mean Age (SD) at 1^st^ voluntary intercourse Age Range at 1st voluntary intercourse	17.16 (2.91) 9-28

^a^Missing geographic data for 18 participants

### Recruitment and procedures

There were two methods of recruitment for this study, online and in-person. Ninety-two percent of the women responded to an online survey, while 8% chose face-to-face participation. There were no significant differences on main outcome measures based on mode of survey administration. Institutional Review Board approved fliers were posted in various community-based organizations, public and private sector businesses, and public and private institutes of higher education in southern California. Permission was given by public and private social and professional listservs that focus on the African American community to provide a brief overview of the study online to recruit participants.

For those who participated via the Internet, a link was provided in the body of the study invitation which directed participants to the survey webpage. Participants for the web-based survey accessed a secure webpage that provided them with a cover letter which included all pertinent information. Participants were asked to indicate that they understood the information *and* that they met the inclusion criteria. Implied consent was used because the survey was anonymous, and signatures were not obtained. A remote address (IP) was returned with submission of the survey responses and examined by the researcher to reduce the likelihood of multiple submissions from a single respondent.

In-person survey participants were given a copy of the implied consent form. The present study was part of a larger study so participants were given a packet of study instruments, but only the data relevant to the concepts identified here are presented. An incentive for participation in this study was the opportunity to win one of three digital audio music players. Participants were only contacted if they were one of the three winners.

## Materials and methods

Sociodemographic information collected included year of birth, education, employment status, household income, geographic region, age at first voluntary intercourse, and relationship status.

### Gender roles

The 24-item Personal Attributes Questionnaire (PAQ) was administered (Spence, Helmreich, & Stapp, 1974). The PAQ is a multidimensional tool that measures three factors: masculinity, femininity, and androgyny. The PAQ is not a global measure of masculinity and femininity, but it has been widely used with diverse populations to provide a classification of individuals in terms of their gender role identity (Bowleg et al., 2000). In responding to the PAQ, participants were asked to choose the best answer that represents the type of person they think they are. Each item consisted of a pair of contradictory characteristics – that is, you cannot be both at the same time. Each extreme response on the subscales was scored 4, the next most extreme scored 3, etc. The feminine/expressive subscale had a Cronbach's alpha of .79. The masculine/instrumental subscale had an original Cronbach's alpha of .59; however, the item *Can make decisions easily/Has difficulty making decisions* was eliminated, and the resulting alpha was .71. Higher scores indicate more traditional gender roles (high masculinity and femininity). Based on low reliability of the androgynous scale (.48), it was eliminated from our analyses.

### Sociosexual orientation

The seven-item SOI (Simpson & Gangestad, 1991) was administered to participants. The tool is designed to measure individual differences in willingness to engage in casual, uncommitted sexual relationships. Three open-ended items asked respondents to report on aspects of their past and future sexual behavior. Three items that assessed attitudinal beliefs were evaluated on a Likert scale (1 = strongly disagree to 9 = strongly agree), and one final item assessed the frequency of sexual fantasies about someone other than the current partner. Higher scores indicated a more unrestricted sociosexual orientation. The Cronbach's alpha was .72.

### Sexual behavior

There were five questions with dichotomous (yes/no) response options to assess participants' past risky sexual behavior: (1) *Have you ever felt obligated to have sex with your partner*? (2) *Have you ever had more than one sexual partner in a 30-day period*? (3) *Have you ever had sex with a partner you thought was having sex with someone else*? (4) *Have you ever had a one-night stand with someone you did not know very well*? and (5) *Have you ever tested positive for a sexually transmitted infection (STI)*?

### Analyses

The analyses were conducted with PASW (version 20.0 for Windows; SPSS Inc., Chicago, IL) statistical software. Descriptive analyses were computed for sociodemographic factors and sexual behaviors. We tested bivariate associations (Table 2) between the SOI total scale and two subscales (PAQ-Masculine and PAQ-Feminine), and independent *t*-tests on the scales of interest and the sexual behavior outcomes (Table 3). Finally, multivariate analyses predicting sexual risk behavior was conducted (Table 4).

**Table 2.** Bivariate associations among predictors.

Variable	SOI	PAQ-Feminine	PAQ-Masculine
SOI	1	−0.150*	−0.048
PAQ-Feminine	−0.150*	1	0.304**
PAQ-Masculine	−0.048	0.304**	1

*p ≤ .05.

**p ≤ .01.

**Table 3.** Independent sample *t*-test comparing sexual risk behaviors by gender roles and sociosexual orientation.

	Obligation to have sex	Greater than one sexual partner in 30 days	Thought partner was having sex with others	Had one-night stand	Previous STI
% Yes					
PAQ-Feminine	23.9 (4.28)	23.3 (4.53)	23.6 (4.20)	23.4 (4.11)	22.9 (4.4)
PAQ-Masculine	21.2 (3.42)	20.7 (3.71)	21.2 (3.68)	21.1 (3.86)	20.9 (3.9)
SOI total	51.1 (26.0)	60.3 (27.1)*	53.5 (26.5)	61.9 (28.3)	55.4 (29.4)*

*p < .05.

**Table 4.** Multivariate logistic regression predicting sexual risk behaviors: ORs (CI).

	OR (CI) Obligation to have sex	OR (CI) Greater than one partner in 30 days	OR (CI) Thought partner having sex with others	OR (CI) One-night stand	OR (CI) Previous STI
PAQ-Feminine	1.043 (0.985–1.104)	1.019 (0.958–1.083)	1.025 (0.962–1.092)	1.013 (0.951–1.079)	0.957 (0.903–1.014)
PAQ-Masculine	1.024 (0.957–1.096)	0.968 (0.900–1.041)	1.050 (0.974–1.131)	1.030 (0.956–1.108)	1.001 (0.935–1.072)
SOI total	1.005 (0.995–1.015)	1.041 (1.027–1.055)**	1.024 (1.011–1.038)**	1.045 (1.031–1.059)**	1.015 (1.005–1.026)*

*p < .05.

**p < .001.

## Results

### Gender roles

Age at first intercourse and education were the only two sociodemographic variables related to the PAQ subscales. A bivariate correlation revealed a positive relationship between age at first intercourse and the PAQ-Masculine subscale (*r* = .18, *p* = .003). Women who reported later voluntary sexual intercourse scored higher on instrumental traits. Education was positively related to the PAQ-Masculine, *F*(5268) = 3.59, *p* < .004, such that women with more formal education also scored higher on instrumental traits. There were no significant associations with sociodemographic variables and the PAQ-Feminine subscale. The two gender role scales were positively correlated with each other (*r* = .30), such that higher scores on the PAQ-Feminine were significantly related to higher scores on the PAQ-Masculine, *F*(1273) = 27.72, *p* < .001.

### Sociosexual orientation

Bivariate correlations revealed significant negative associations SOI and age (*r* = −.17, *p* = .005), and age at first intercourse (*r* = −.17, *p* = .005). Younger participants, and those who were younger the first time they voluntarily engaged in sexual intercourse had higher scores on the SOI. An Analysis of variance revealed a significant difference in scores on the SOI for women, based on relationship status *F*(2269) = 7.91, *p* < .001. *Post hoc* tests reveal that women in casual relationships (*n* = 47, 61.38) had higher scores than those in committed relationships (*n* = 180, 45.94), *p* = .001.

### Research questions

Linear regression analyses revealed that gender roles and sociosexuality were significantly related to each other. The PAQ-Feminine scale was negatively related to the SOI scale, *F*(1273) = 6.26, *p* = .013. Women with lower scores on the SOI (e.g. more restrictive) saw themselves as having more expressive traits. The PAQ-Masculine scale was not significantly related to the SOI.

Multivariate logistic regression analyses are presented in Table 4. SOI was related to four of the five sexual behavior outcomes. The higher a woman scored on the SOI scale, the more likely she was to report having sex with more than one partner in a 30-day period (OR 1.041; CI 1.027–1.055, *p* < .001), to have had sex with a man she knew was having sex with another person (OR 1.024; CI 1.011–1.038, *p* < .001), to have had a one-night stand with someone she did not know very well (OR 1.045; CI 1.031–1.059, *p* < .001), and to have had an STI (OR 1.015; CI 1.005–1.026, *p* = .004). For every 10-point increase on the SOI scale, thus a more unrestricted sociosexuality (i.e. comfortable engaging in sex without commitment or emotional closeness) the odds of these four sexual risk behaviors will increase among participants by 49% for having more than one partner in 30 days; 27% for having a partner thought to be having sex with others; 55% for having a one-night stand; and 16% for having a previous STI. Neither the PAQ-Feminine or PAQ-Masculine scales were related to any of the sexual behavior outcome variables.

## Discussion

The purpose of this study was to examine the relationship between gender roles and sociosexuality, and to explore their predictive patterns of sexual risk behavior among adult, heterosexual Black women. A number of findings from this study should be highlighted. Gender role was related to certain sociodemographic variables and sociosexuality; however it was not significantly related to any of the predicted sexual behavior outcomes. Education emerged as a related correlate of the PAQ-Masculine subscale, but not the PAQ-Feminine subscale. We found that education increased with embodiment of more instrumental characteristics. Earlier work found that higher levels of education were related to fewer expressive traits for both men and women (Harris & Firestone, 1998). Bowleg et al. (2000) showed that more education for African American women was related to more instrumental traits (i.e. masculine) and expressive traits (i.e. feminine). One thought about our results is that the more time a woman spends pursuing education and/or participating in the workforce, the more instrumental traits she may express. According to a previous study, education provides greater exposure to nontraditional gender roles and fewer acceptances of traditional stereotypes and myths related to gender and roles (Davis & Greenstein, 2009).

We found that those women who scored higher on the PAQ-Feminine subscale were more restrictive in their SOI scores. There is a lack of information on sociosexuality and gender role among women; however, our finding is consistent with previous research done on women by Mikach and Bailey (1999). Our results are also in line with research that suggests women who internalize traditional expressive roles may limit sexual partners to only those who are thought to be long-term – which is consistent with the ideology behind sociosexuality (Okami & Shackelford, 2001). It is a curious finding that the PAQ-Feminine subscale was not related to any of the sexual behavior outcomes. Previous studies indicate that conforming to traditional roles, in the form of hyperfemininity, may be related to the likelihood of multiple sexual partners and/or concurrent sexual relationships (Murnen & Byme, 1991). If restrictive sociosexuality is related to less permissive sexual attitudes and behaviors, and more insistence of intimacy and commitment in a relationship prior to sex, then it, theoretically, should be negatively related to our risky sexual behaviors.

Much of the literature focused on gender roles and sexual behaviors reveal a significant relationship between the two among African American women. One explanation for our results may be related to a lack of focus on other factors that may be mediating, or moderating, this relationship. One such factor is the gender disparity that exists in the African American community. This disparity posits that there are more “available” men than women to engage in intimate relationships. This imbalance has been shown to significantly impact the sexual behavior of some African American women (Bontempi, Eng, & Quinn, 2008; Ferguson, Quinn, Eng, & Sandelowski, 2006) in ways such as altering their preventative behaviors because of fear of losing a relationship or not being able to find one (El-Bassel, Caldeira, Ruglass, & Gilbert, 2009). We are not suggesting that African American women only date African American men; however, the rate of interracial marriages between African American women and men of other races/ethnicities is low (Childs, 2005). Another plausible explanation for the lack of significance between gender role and risky sexual behaviors is that behaviors we examined may not be seen in the same light as condom use or condom negotiation, which much of the literature focuses on. We believe that future studies examining gender roles and sexual behavior should include more variety in the types of sexual behaviors explored. There is a plethora of behaviors that put women at risk for contracting and transmitting HIV other than condom use. Finally, a contributing factor to the lack of significance could be that the mean scores on the PAQ-Masculine and PAQ-Feminine subscales were close (21.2 and 23.9, respectively) and the two subscales were highly correlated. The scales were thought to be orthogonal which should measure two different categories of traits (Spence et al., 1974). Since gender role attitudes and beliefs are socially and culturally developed, the use of the descriptors may be too simplistic for the experience of more contemporary African American women. The authors are in agreement with Nguyen et al. (2010) in asserting that future research should seek to identify a new measurement that uses a more holistic approach that reflects the experiences and emically defined characteristics important to African American women.

Finally, we found that sociosexuality was related to four of the five sexual behavior outcome variables. Sociosexuality has been shown to be a significant predictor of various risky sexual behaviors (Diaz-Loving & Rodriguez, 2008; Hall & Witherspoon, 2011; Simpson & Gangestad, 1991; Yost & Zurbriggen, 2006). Our results were consistent with previous studies, in that scores on the SOI were significantly related to increased odds that women engaged in the following risky sexual behaviors: (1) multiple partners in a 30-day period; (2) sex with someone who they thought may be having sex with someone else; (3) a one-night stand with someone they do not know well; and (4) tested positive for an STI.

There are few studies that focus on sociosexuality and its relationship with risky sexual behavior within racial/ethnic minority populations. One of these studies found unrestricted sociosexuality was significantly related to risky sexual behavior such as increased sexual contact, sexual seduction, and an increased number of sexual partners among 209 heterosexual Mexican adults (Diaz-Loving & Rodriguez, 2008). Another study with 57 heterosexual African American college students found that higher levels of sociosexuality was significantly related to engagement in riskier sexual behaviors such as unprotected vaginal and anal intercourse, multiple sexual partners, substance use prior to or during sexual encounters, and STI (Hall & Witherspoon, 2011). The phenotypic manifestations of sociosexuality may change over the life span. During the dating years, individual differences in sociosexuality might be most clearly revealed in differential willingness to engage in sex prior to the development of commitment and strong emotional ties. During the marital years, sociosexuality might be most clearly evident in either differential willingness to remain in unsatisfactory marital relationships or differential susceptibility to being drawn out of such relationships by attractive alternative partners (Jones, 1998; Simpson & Gangestad, 1991). Sociosexuality is an important individual difference variable and more attention needs to focus on the sociosexual attitudes and behaviors of African American women, and its potential relationship to HIV risk-related sexual behavior.

### Limitations and strengths

One limitation may be the use of self-report for sexual behavior information. It is always possible that participants are not able to accurately recall their thoughts, attitudes, and behaviors. It is possible that participants underestimated or overestimated their responses and/or experiences. As with all non-experimental studies, predictive relationships can only be assessed. As such, causal statements about psychosocial correlates that predict sexual behavior cannot be made based on the associations established by this study. Gender, gender role, and race/ethnicity are inextricably linked, therefore future investigations should explore questions that address these different areas and how they are associated with each other. Along with the limitations, this study had a number of strengths. One of the greatest strengths of this study is the diversity of the African American women. Having a diverse representation, geographically and socioeconomically, helps provide data that may be used in prevention and intervention planning efforts. Many of the studies on African American women and sexual behavior use comparison groups. Our study was designed to examine the within-group variations that are often overlooked.

## Conclusion

Some debate the role of individual-level psychosocial factors in sexual risk behaviors, given the established body of literature accounting for social and environmental determinants of HIV (CDC, 2010a). Understanding the dimensions of personality and how personalities are shaped from an ecological perspective is warranted. Both individual and environmental level factors influence health behaviors and health outcomes (CDC, 2010a; McLeroy, Bibeau, Steckler, & Glanz, 1988). To this end, gender-specific multi-level interventions need to be developed, adopted, and disseminated widely to strengthen Black women's capacity to engage in safer sex practices (Bartholomew, Parcel, Kok, Gottlieb, & Fernandez, 2011; Rogers, 1995). There is a growing community-wide misconception that only the poor and uneducated engage in high-risk sexual intercourse. Although prevention and intervention services in low-income communities are imperative (CDC, 2010b; Denning & DiNenno, 2010) our data illustrates a continuum of sociosexual orientation coupled with self-reported sexual risk behaviors among a highly educated demographic of Black women. Interventions tailored to the needs of economically diverse Black women exhibiting a willingness to engage in casual, uncommitted sex would contribute substantially to the knowledge base. To our knowledge, this is one of few studies to address the limited data on the role of sociosexuality in HIV-related sexual risk behavior. Because these personality traits have been demonstrated in the current study to contribute signifıcantly to four sexual risk behaviors, efforts to continue examining the nuances of these factors could highlight barriers to HIV preventative behaviors and inform interventions.

## Note

1. African American and Black will be interchanged throughout the manuscript based on how information is reported in sources cited.

## References

[CIT0001] Amaro H. (1995). Love, sex, power: Considering women's realities in HIV prevention. *American Psychologist*.

[CIT0002] Bartholomew L. K., Parcel G. S., Kok G., Gottlieb N. H., Fernandez M. E. (2011). *Planning health promotion programs: An intervention mapping approach*.

[CIT0003] Binion V. J. (1990). Psychological androgyny: A Black female perspective. *Sex Role*.

[CIT0004] Bird S. T., Harvey S. M. (2001). No glove, no love: Cultural beliefs of African American women regarding influencing strategies for condom use. *Internationally Quarterly of Community Health Education*.

[CIT0005] Bontempi J. M., Eng T., Quinn S. C. (2008). Our men are grinding out: A qualitative examination of sex ratio imbalances, relationship power, and low-income African American women's health. *Women's Health*.

[CIT0006] Bowleg L., Belgrave F. Z., Reisen C. A. (2000). Gender roles, power strategies, and precautionary sexual self-efficacy: Implications for Black and Latina women's HIV/AIDS protective behaviors. *Sex Roles*.

[CIT0007] Campbell C. A. (1995). Male gender roles and sexuality: Implications for women's AIDS risk and prevention. *Social Science and Medicine*.

[CIT0008] Centers for Disease Control and Prevention. (2010a). http://www.cdc.gov/socialdeterminants/docs/SDH-White-Paper-2010.pdf.

[CIT0009] Centers for Disease Control and Prevention. (2010b). http://www.cdc.gov/nchhstp/newsroom/povertyandhivpressrelease.html.

[CIT0010] Centers for Disease Control and Prevention. (2011). *Fact sheets: HIV among African Americans*.

[CIT0012] Childs E. C. (2005). Looking behind the stereotypes of the “Angry Black Woman”: An exploration of Black women's responses to interracial relationships. *Gender and Society*.

[CIT0013] Cooper M. L., Agocha V. B., Sheldon M. S. (2000). A motivational perspective on risky behaviors: The role of personality and affect regulatory processes. *Journal of Personality*.

[CIT0015] Davis S. N., Greenstein T. N. (2009). Gender ideology: Components, predictors, and consequences. *Annual Review of Sociology*.

[CIT0016] Denning P., DiNenno E. (2010). http://www.cdc.gov/hiv/topics/surveillance/resources/other/pdf/poverty_poster.pdf.

[CIT0017] Diaz-Loving R., Rodriguez G. G. (2008). Sociosexual orientation and sexual behavior in Mexican adults. *Social and Personality Psychology Compass*.

[CIT0018] Ehrhardt A. A., Wasserheit J. N. (1991). Age, gender and sexual risk behaviors for sexually transmitted diseases in the United States. K. K. Holmes & S. Aral (Eds.), *Research issues in human behavior and sexually transmitted diseases in the aids era*.

[CIT0019] El-Bassel N., Caldeira N. A., Ruglass L. M., Gilbert L. (2009). Addressing the unique needs of African American women in HIV prevention. *American Journal of Public Health*.

[CIT0020] Ferguson Y. O., Quinn S. C., Eng E., Sandelowski M. (2006). The gender ratio imbalance and its relationship to risk of HIV/AIDS among African American women at historically Black colleges and universities. *AIDS Care*.

[CIT0022] Hall N. M., Witherspoon D. D. (2011). The influence of sociosexuality and perceived susceptibility on the sexual behavior of African American college students. *Journal of Best Practices in Health Professions Diversity: Research, Education, and Policy*.

[CIT0023] Harris R. J., Firestone J. M. (1998). Changes in predictors of gender role ideologies: A multivariate analysis. *Sex Roles*.

[CIT0024] HIV Prevention Trials Network. (2012). http://hptn.org/web%20documents/HPTN064/064_factsheet_29Feb2012.pdf.

[CIT0025] Jarama S. L., Belgrave F. Z., Bradford J., Young M., Honnold J. A. (2007). Family, cultural and gender role aspects in the context of HIV risk among African American women of unidentified HIV status: An exploratory qualitative study. *AIDS Care*.

[CIT0027] Kaiser Family Foundation. (2012). *Black Americans and HIV/AIDS*.

[CIT0028] Kerrigan D., Andrinopoulous K., Johnson R., Parham P., Thomas R., Ellen J. M. (2007). Staying strong: Gender ideologies among African-American adolescents and the implications for HIV/STI prevention. *Journal of Sex Research*.

[CIT0029] Kinsey A. C., Pomeroy W. B., Martin C. E. (1948). *Sexual behavior in the human male*.

[CIT0030] Leech T. G. J. (2010). Everything's better in moderation: Young women's gender role attitudes and risky sexual behavior. *Journal of Adolescent Health*.

[CIT0031] Littlefield M. B. (2003). Gender role identity and stress in African American women. *Journal of Human Behavior in the Social Environment*.

[CIT0032] McLeroy K. R., Bibeau D., Steckler A., Glanz K. D. (1988). An ecological perspective on health promotion programs. *Health Education Quarterly*.

[CIT0034] Murnen S., Byme D. (1991). Hyperfemininity: Measurement and initial validation of the construct. *Journal of Sex Research*.

[CIT0035] Nguyen A. B., Clark T. T., Hood K. B., Corneille M. A., Fitzgerald A. Y., Belgrave F. Z. (2010). Beyond traditional gender roles and identity: Does reconceptualization better predict condom-related outcomes for African American women?. *Culture, Health & Sexuality*.

[CIT0036] Okami P., Shackelford T. (2001). Human sex differences in sexual psychology and behavior. *Annual Review of Sex Research*.

[CIT0037] Ostovich J. M., Sabini J. (2004). How are sociosexuality, sex drive, and lifetime number of sexual partners related?. *Personality and Social Psychology Bulletin*.

[CIT0038] Pleck J. H., Sonenstein F. L., Ku L. C. (1993). Masculinity ideology: Its impact on adolescent males’ heterosexual relationships. *Journal of Social Issues*.

[CIT0039] Rogers E. M. (1995). *Diffusion of innovations*.

[CIT0040] Shearer C. L., Hosterman S. J., Gillen M. M., Lefkowitz E. S. (2005). Are traditional gender role attitudes associated with risky sexual behavior and condom-related beliefs?. *Sex Roles*.

[CIT0041] Sikkema K. J., Wagner L. I., Bogart L. M., Eisler M. R., Hersen M. (2000). Gender and cultural factors in the prevention of HIV infection among women. *Handbook of gender, culture and health*.

[CIT0042] Simpson J. A., Gangestad S. W. (1991). Individual differences in sociosexuality: Evidence for convergent and discriminant validity. *Journal of Personality and Social Psychology*.

[CIT0043] Spence J. T., Helmreich R., Stapp J. (1974). The personal attributes questionnaire: A measure of sex-role stereotypes and masculinity–femininity. *Journal Supplement Abstract Service: Catalog of Selected Documents in Psychology*.

[CIT0044] Westerlund M., Santilla P., Johannson A., Varjonen M., Witting K., Jem P. (2010). Does unrestrictive sociosexual behaviour have a shared genetic basis with sexual coercion?. *Psychology, Crime & Law*.

[CIT0045] Wingood G. M., DiClemente R. J. (1997). The effects of an abusive primary partner on the condom use and sexual negotiation practices of African American women. *American Journal of Public Health*.

[CIT0046] Yost M. R., Zurbriggen E. L. (2006). Gender differences in the enactment of sociosexuality: An examination of implicit social motives, sexual fantasies, coercive sexual attitudes, and aggressive sexual behavior. *The Journal of Sex Research*.

